# Epidemiology of Cancers in Kashmir, India: An Analysis of Hospital Data

**DOI:** 10.1155/2016/1896761

**Published:** 2016-07-05

**Authors:** Mariya A. Qurieshi, S. M. Salim Khan, Muneer A. Masoodi, Uruj Qurieshi, Quratul Ain, Yasmeen Jan, Inaamul Haq, Sheikh Zahoor Ahmad

**Affiliations:** ^1^Department of Community Medicine, Government Medical College, Srinagar, Kashmir 190010, IndiaIndia; ^2^Department of Surgical Oncology, SKIMS, Srinagar, Kashmir 190011, India

## Abstract

Cancer is a leading cause of mortality and morbidity in the world. The aim of the present study was to measure the pattern of different cancers in Kashmir, India, a cancer belt with peculiar cancer profile. A hospital based cancer registry was started by the Department of Community Medicine, Government Medical College, Srinagar, in January 2006, wherein information was collected from cancer patients who were diagnosed and treated in the hospital. Data has been analysed for a period extending from January 2006 to December 2012. Descriptive analysis has been done by using statistical software. A total of 1598 cancer patients were admitted during this period. Overall male to female ratio was 1.33 : 1. Stomach cancer was the most commonly reported cancer (25.2%), followed by colorectal cancer (16.4%) and lung cancer (13.2%) among males. For females, colorectal cancer (16.8%), breast cancer (16.1%), and stomach cancer (10.4%) were the most frequently reported cancers in order of frequency. Tobacco related cancers contributed to more than three-fourths of cancers among men and more than half of cancers for women. There is an urgent need to set up a population based cancer registration system to understand the profile of cancers specific to this geographic region.

## 1. Introduction

World is facing an epidemic of noncommunicable diseases and it is believed to get worse at the end of this decade. Noncommunicable diseases are responsible for more than three-fifths of the deaths globally (36 million), largely contributed by cardiovascular diseases (48% of noncommunicable disease deaths), cancers (21% of noncommunicable disease deaths), chronic respiratory diseases (4.2 million deaths), and diabetes mellitus (1.3 million deaths) [1].

It is predicted that cancer will be an important cause of mortality and morbidity all over the world in next few decades. Further, it is estimated that the incidence of new cancer cases will rise from 12.7 million cases in 2008 to 21.4 million by 2030, and nearly two-thirds of it will occur in low-to-middle income countries [2]. However, these predictions and estimations are based on the countries which have their own cancer registry. Unfortunately, information on the number of new cancer cases and cancer deaths is not available from major regions of the world and according to World Cancer Report 2008 less than 20% of the world's population was covered by cancer registration in year 2000. Moreover, the coverage was unequal, as in contrast to Latin America, where 95% of the population was covered, in Asia it was only 9% [3]. In order to identify the burden of cancers in India, a Cancer Registry Programme was started by Indian Council of Medical Research in 1982 with an objective of determining the magnitude and pattern of various cancers. The programme started with six cancer registries, of which three were population based cancer registries (PBCRs) and three were hospital based cancer registries (HBCRs). It has gradually expanded over the years and at present there are 24 population based cancer registries and 5 hospital based cancer registries working under the network of National Cancer Registry Programme. Lately in 2005-2006, Northeastern states were also covered in the registry programme. Unfortunately, none of the states from Northern India have Cancer Registry Programme.

Kashmir is a landlocked geographical entity located at a very high altitude, with a multiethnic Muslim majority population with unique cultural practices. It has been traditionally considered to be an endemic cancer zone with peculiar cancer profile [4]. There is no population based cancer registry in place in Kashmir which would have provided us with a more holistic perspective of magnitude of cancer burden here. Till date, there have been a number of published reports of cancer in Kashmir, almost all of which have been single institute hospital based studies. With this backdrop, in 2005, the Department of Community Medicine of Government Medical College Srinagar of Jammu and Kashmir state took up the lead in starting its own hospital based cancer registry with an objective of assessing pattern and distribution of various cancers among the patients attending the hospital. SMHS hospital is centrally located associated hospital of Government Medical College, Srinagar, which provides speciality care to the needy patients. In addition, superspeciality services are also provided by the hospital especially to cancer patients. Cancer services are offered by the Departments of Surgery, Medicine, Otorhinolaryngology, Radiotherapy, Gynaecology and Obstetrics, and Orthopaedics. The hospital has its own Radiation Oncology Department which exists in the absence of specialised Medical and Surgical Oncology Departments. Even though the hospital is not a designated cancer centre, a major chunk of patients of Kashmir division including Ladakh and neighbouring districts of Jammu division of J&K state seek cancer treatment here, as it is one of the two major tertiary care government hospitals in Kashmir division of Jammu and Kashmir state. Moreover, many patients are directly referred to this hospital for radiation treatment from private sector also.

## 2. Methods

The present study was carried out in one of the oldest tertiary care hospitals which is located in the central district of Kashmir valley. Shri Maharaja Hari Singh (SMHS) hospital is a teaching hospital which provides both speciality and superspeciality services. It has four associated speciality hospitals which are also located in the district of Srinagar and provide paediatric, orthopaedic, and respiratory medicine and gynaecology and obstetrics services. This hospital, however, caters to the health needs of the people not only from the Kashmir division but from Ladakh and Kargil as well. In the present paper, an effort has been made to project the magnitude and pattern of various cancers being treated in SMHS and its associated hospitals from January 2006 to December 2012. During 2010 and 2011 completeness of data collection could not be ensured due to political disturbances. A cancer data collection instrument was designed which was pretested initially. Information from cancer patients who were admitted in the hospitals was collected. For this purpose, medical interns were trained to administer the study instrument to maintain the reliability of the information collected. They made regular visits to the in-house patients in SMHS and its associated hospitals. They located the newly diagnosed cancer cases including suspected cases and collected demographic information, details of diagnosis, and treatment modality provided. Only those cases which were histologically proven were included. For suspected cases, where cancer was not confirmed, they were excluded from the study after undertaking detailed investigation as per the protocol. Duplication of data was taken care of at two levels, firstly by assigning a code to each case which is done by the hospital registration and records section and secondly at the level of data entry. The information was then pooled and processed and coding was done according to International Classification of Diseases for Oncology (ICD-O-3 and ICD 10).

## 3. Results

Total cancer patients registered in the cancer registry were 1598 (January 2006–December 2012), of which 914 (57.2%) were males and 684 (42.8%) were females. Sex distribution of cancers across different age groups is shown in Table 1. Most of the patients were in the age group of 65–74 years for males and 55–64 years for females. Overall male to female ratio was 1.33 : 1. Two-thirds of the cancer patients belonged to age group of 45 to 74 years. Nearly half of the patients had cancer of gastrointestinal tract (45.4%) followed by respiratory tract (14.6%) and female genital organs (8.6%). Breast and skeletal cancers comprised 6.9% and 5.6% of all cancers, respectively. Cancers involving haematolymphoid, urinary tract, endocrine, soft tissue, male genital organs, and unknown primary site contributed 18.9% of all cancers.

Gastric cancer was the most commonly encountered cancer (18.8%) followed by colorectal cancer (16.6%), lung cancer (9%), head and neck cancer (7.9%), and breast cancer (6.9%). Among men five most common cancer sites included gastric cancer (25.2%), colorectal cancer (16.4%), lung cancer (13.2%), head and neck cancer (10.8%), and oesophageal cancer (5.4%). In women, colorectal cancer (16.8%) was the most commonly reported cancer followed by breast cancer (16.1%), gastric cancer (10.4%), ovary cancer (9.8%), and thyroid cancer (7.5%). Cancers of oral cavity, pharynx, larynx, nose, nasal cavity, paranasal sinuses, and salivary glands have been grouped under head and neck cancers. Among head and neck cancers, laryngeal cancer is the most commonly reported one contributing to 58% of all head and neck cancers (Table 2).

**Figure 1:**
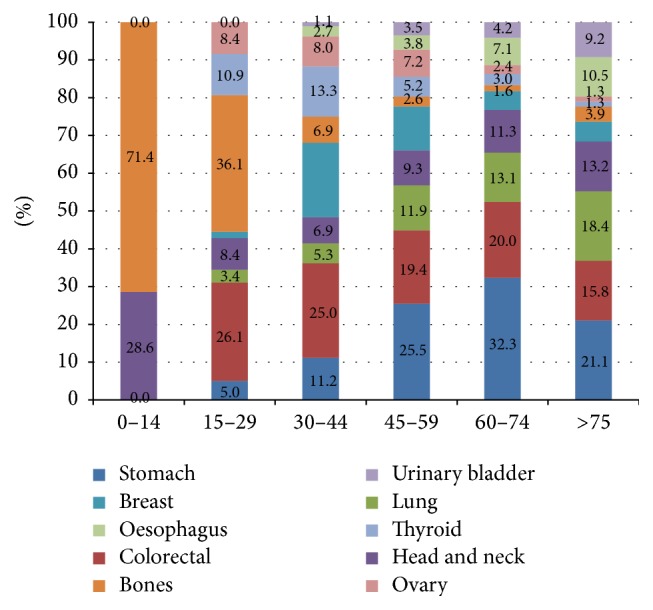
Proportion of common cancers across different age groups.

Skeletal malignancies were the most common of all cancers up to <30 years of age accounting for 71.4% of all cancers in the age group of 0–14 years and 36.1% of all cancers in the age group of 15–29 years. In the age group of 30–44 years colorectal cancer topped the list (25%) followed by cancers of the breast (19.7%) and thyroid (13.3%). Beyond 44 years of age gastric cancer was the commonest. Colorectal cancer and lung cancer were the second and third most common cancers between 45 and 74 years, while in the older age groups the positions reversed with lung cancer being reported more commonly (18.4%) as compared to colorectal cancer (15.8%) (Figure 1).

**Figure 2:**
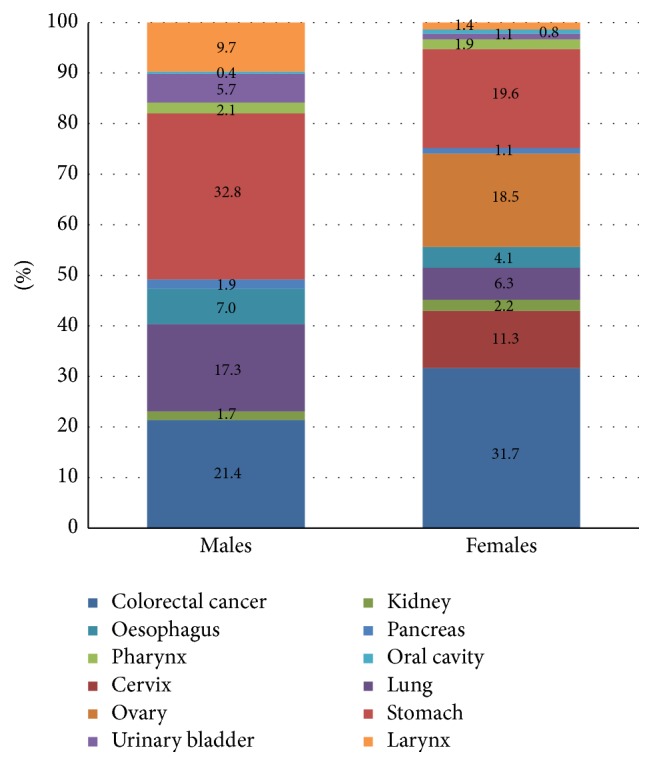
Stack (100%) diagram showing proportion of specific tobacco related sites relative to all tobacco related cancers (2005–2012).

In the study population 55.2% (505/914) and 14.6% (100/684) of males and females (*n* = 1277) were ever smokers, respectively. Overall and sex differences in the relative proportion of TRCs (2005–2012) were also calculated. The proportion of tobacco related cancers in our study is 66.6%. Among males 76.7% of cancers were tobacco related cancers as compared to 53.1% in females. Among males major sites contributing to total TRCs were gastric (32.8%), colorectal (21.4%), lung (17.3%), and larynx (9.7%); and for females they were colorectal (31.7%), gastric (19.6%), cervix (19.6%), and ovary (18.5%). The proportion of sites of cancer associated with tobacco use in males and females relative to all tobacco related cancers is shown in Figure 2.

## 4. Discussion

Measuring the magnitude and distribution of various cancers in community is imperative to ensuring well informed policies on cancer care and prioritizing resource allocation. Further robust data is needed to inform medical care with highest level of evidence. Hence, cancer registries are considered to be integral part of cancer management in any part of the world. Kashmir is a landlocked mountainous area with unique tradition, culture, and dietary habits. The magnitude and profile of cancer in Kashmir are poorly reported; however, gastrointestinal cancers are reportedly believed to be the most common. Earliest scientific reporting of cancer from Kashmir dates back to 1866, when Elmslie reported 30 cases of cutaneous epithelioma (which is now known as Kangri cancer) [5]. This was followed by further reports by others [6–8]. The reports from these times established Kangri cancer as the most common cancer of that era.

In the present study more men were reported to have cancer as compared to women with a ratio of 1.33 : 1. This was true for all the commonly encountered cancers in our population like gastric cancer (3.24 : 1), colorectal cancer (1.3 : 1), head and neck cancer (3.54 : 1), bone cancer (1.17 : 1), and lung cancer (5.26 : 1), except thyroid cancer, where the ratio was reversed (1 : 2.3). The gender disparity in case of thyroid cancers is a well-established fact owing to hormonal influence [9]. This study highlighted that the gastric cancer is the leading cancer encountered in our hospital (18.8%) and the leading cancer site among men (25.2%) and third most common cancer among females (10.4%). Gastric cancer is one of the most common cancers in Kashmir along with oesophageal cancer. Both of them account for almost 25% to 34% of the cancers in this population [4, 10, 11]. Such observation may be due to the fact that majority of the risk factors that have been incriminated in the causal pathway of gastric cancer are prevalent in Kashmir. Role of diet, smoking,* H. pylori* infection, and genetic susceptibility of host have been widely studied throughout the world [12]. Role of salted tea, commonly known as noon chai, which is a peculiar beverage of Kashmiri and is prepared and consumed by all sections of the population, has been proposed and widely studied as a risk factor [13]. In addition, most of the other dietary risk factors for gastric cancer like pickled food, high rice intake, spicy food, excess chilly consumption, and intake of food at high temperature which have emerged as significant risk factors and have been studied in other parts of India are prevalent in Kashmir as well [14–16]. Other than dietary factors, use of tobacco in different forms like cigarette smoking, hukka, and snuff is quite prevalent in Kashmir. Tobacco use in the form of hukka also known as hubble bubble and snuff is quite prevalent in rural Kashmir. Few preliminary studies using small sample size have been done to study the role of gene polymorphism and gastric cancer which have shown a link between gene polymorphism and gastric cancer in Kashmiri population [17]. However, it needs to be studied further in detail. We observed colorectal cancer as the most common cancer among females (16.8%) and second most common cancer among males (16.4%) with M : F ratio of 1.3 : 1. Globally also colorectal cancer is the third most common cancer reported among females and second most common cancer for males [18]. This may be due to the fact that there has been changing trend in the consumption of type of food intake by a typical Kashmiri over past few decades. A shift from intake of traditional food which was rich in complex carbohydrate to more of simple carbohydrates has increased the risk of obesity. It is well-established fact in the literature that westernisation such as obesity and physical inactivity increases the incidence of colorectal cancer in many countries [19]. Breast was the second most common site involved among females (16.1%). Our observation is comparable with the results of population based cancer registries of National Cancer Registry Programme in India, where they have reported breast cancer as the leading cancer among females of Mumbai, Delhi, Bangalore, Bhopal, Ahmadabad, Kolkata, and Chennai [20]. Lung cancer is the third most common cancer reported overall (9%) and also third most common cancer reported among males (13.2%). The reason for this may be due to the fact that use of tobacco in the form of smoking is quite prevalent among Kashmiri males which has been shown by earlier studies conducted in Kashmir [21, 22]. Lung cancer has been also reported to be the most common cancer among males in India from four PBCRs [20].

A striking and a contrasting observation made here was the very low prevalence of cervical cancer among females contributing to mere 6% of all cancers reported among females. When we compare it with rest of India, cervical cancer is second only to breast cancer and at some places it ranks as the most common cancer among Indian females [20]. We could not find any study in the literature done previously in Kashmir to know the prevalence of cervical cancer which is the leading cause of mortality and morbidity among females not only worldwide but also in India. Only one population based study has been done in a rural district of Kashmir, wherein females were screened for cervical cancer and it was observed that none of the screened females showed evidence of any grade of dysplasia or cervical cancer [23]. Most plausible reason for this could be male circumcision and absence of risk factor like sexual promiscuity, both of which have been implicated in the direct acquisition of infection by oncogenic strains of Human Papilloma Virus (HPV) [24]. Head and neck cancer was the fourth most common cancer overall (7.9%) and also fourth most common cancer among men (6.9%) with M : F ratio of 3.54 : 1. When we compare different sites of head and neck involved in our population with rest of India, a contrasting difference can be noticed. In rest of India oral cancer ranks among the top three of all cancers owing to the habit of chewing tobacco, a practice not observed in Kashmir [25]. On the contrary, in our case, oral cancers constitute only 6.6% of head and neck cancers against laryngeal cancers which contribute to 58.2% of all head and neck cancers. The other reason for this can be the fact that tobacco use in the form of smoking is common among men, which is a known risk factor for laryngeal cancer [26].

The relative proportion of cancers associated with the use of tobacco has been analysed according to the latest monograph of International Agency for Research on Cancer. International Agency for Research on Cancer (IARC) has enlisted certain anatomical sites which are associated with tobacco use. IARC in 1987 reported that there is association between tobacco use and cancer of lip, tongue, mouth, pharynx, oesophagus, larynx, lung, and urinary bladder. Later on, in 2004, in a newer monograph, it is stated that there is a sufficient evidence to establish a causal association between use of tobacco and cancers of nasal cavity, uterine cervix, oesophagus (adenocarcinoma), stomach, liver, kidney, and myeloid leukaemia apart from the sites already mentioned in the previous monograph (IARC, 1987). The latest monograph (2004) has added ovary, colon, and rectum to its list [27].

Tobacco related cancers (TRCs) contributed to two-thirds of all cancers with three-fourths of cancers among men and more than half of cancers among women which is more in comparison with the data available from population based cancer registries in India, where they have shown that TRCs contribute to nearly half of all cancers among males and only one-fifth of cancers among females. This difference can be attributed to the fact that their classification of TRCs is based on IARC monograph in 1987 which includes only few sites and we have used latest classification.

In recent times, very few papers have reported the magnitude and distribution of cancer in Kashmiri population and unfortunately all of them are derived from hospital based data [4, 10, 11, 21]. In the report published by Dhar et al., data from single department (Radiation Oncology) covering three years (1986–88) was depicted, which is far from true picture prevalent in community [21]. Ayub et al. have attempted to give a more realistic scenario of cancer problem in Kashmir. They have combined the data of two sole tertiary care hospitals (SKIMS and SMHS) serving Kashmir valley. However, they have reported on data procured from only two departments, namely, Radiation Oncology Departments of both hospitals. Since major chunk of patients in either hospital are treated in other departments without ever being referred to Radiation Oncology Department, they are never registered in these departments and hence have been missed in their report [11]. The study published by Pandith and Siddiqi suffers from similar problem, in being single institutional data [10]. The data published by Rasool et al. is a better depiction of hospital cancer workload, as it has been procured from hospital based cancer registry where the chance of missing a cancer patient having come in contact with the hospital is the least. Nonetheless it cannot claim to give true picture of the problem present in community [4].

The present study is probably the first study of its kind which has depicted the true caseload of cancer being handled in SMHS hospital. We have collected the data regarding newly diagnosed cases of cancer from all departments involved in management of cancers over a defined period of time. However, it cannot be ruled out that still a few patients might have been missed out especially those who might have been evaluated on OPD basis and subsequently they might have shifted to some other hospital for further treatment. Nonetheless this data in no manner portrays the true picture of cancer in Kashmir valley and it suffers from the same deficiencies as the previous studies in being purely hospital based data.

## 5. Conclusion 

Keeping in view the diversified culture of India, it would be worthwhile to study the peculiar pattern of cancers in Kashmir. For this a reliable and a valid population based cancer registration system should be established in this northern state of India as is in other states of India on priority as much of the cancer statistics available from Kashmir is based on hospital data which is compromised by its limited coverage and expert services available as is evident from the cancer research done previously in Kashmir.

## Additional Points

Even though the present study is giving us an idea about the pattern of cancers treated at SMHS hospital, it is far from the true picture prevalent in the community. In year 2010-11 the completeness of data collection could not be ensured due to political disturbance.

## Supplementary Material

Supplementary material is in response to the editors query on detailed breakdown of all cancers analysed during the study period.

## Figures and Tables

**Table 1:** Age-sex distribution of cancers (*n* = 1556)^*∗*^.

Age group	Males	Females
0–14	16 (1.03%)	9 (0.58%)
15–24	42 (2.70%)	53 (3.41%)
25–34	47 (3.02%)	63 (4.05%)
35–44	76 (4.88%)	114 (7.33%)
45–54	137 (8.80%)	141 (9.06%)
55–64	249 (16.00%)	152 (9.77%)
65–74	255 (16.39%)	100 (6.43%)
≥75	72 (4.63%)	30 (1.93%)

Total	894 (57.5%)	662 (42.5%)

^*∗*^Missing data for 42.

**Table 2:** Ten most common cancers (overall and across sex).

Sites	Overall	Males		Females	
	*n* = 1598	*n* = 914		*n* = 684	
Stomach	301 (18.8)	Gastric	230 (25.2)	Colorectal	115 (16.8)
Colorectal	265 (16.6)	Colorectal	150 (16.4)	Breast	110 (16.1)
Lung	144 (9)	Lung	121 (13.2)	Gastric	71 (10.4)
Head and neck	127 (7.9)	Head and neck	99 (10.8)	Ovary	67 (9.8)
Breast	110 (6.9)	Oesophagus	49 (5.4)	Thyroid	51 (7.5)
Bones	89 (5.6)	Bone	48 (5.3)	Cervix	41 (6)
Thyroid	73 (4.6)	Urinary bladder	40 (2.5)	Bone	41 (6)
Ovary	67 (4.2)	Skin	28 (3.1)	Head and neck	28 (4.1)
Oesophagus	64 (4)	Thyroid	22 (2.4)	Uterus	27 (3.9)
Urinary bladder	44 (2.8)	Testes	15 (1.6)	Lung	23 (3.4)
Others	314 (19.6)	Others	112 (12.2)	Others	110 (16.1)
