# Supplementary material for: Epidemiology of Cancers in Kashmir, India: An Analysis of Hospital Data
Source: Adv Prev Med. 2016 Jul 5;2016:1896761. doi: 10.1155/2016/1896761 (PMC4949346; doi:10.1155/2016/1896761)
Supplement: Supplementary file 1 — Supplementary material is in response to the editors query on detailed breakdown of all cancers analysed during the study period. [file 1896761.f1.docx]

| **ICD-10** | **Cancer Sites** | **#** | % |
| --- | --- | --- | --- |
| C00-C26.9 | **Digestive System** | **726** | **45.43** |
| C 02 | Tongue | 4 | 0.25 |
| C 06.9 | Oral cavity | 6 | 0.37 |
| C 07 | Parotid gland | 10 | 0.62 |
| C 08.0 | Submandibular gland | 5 | 0.31 |
| C 13.0 | Hypopharynx | 6 | 0.37 |
| C 14.0 | Oropharynx | 6 | 0.37 |
| C 15 | Oesophagus | 31 | 1.94 |
| C 16 | Gastroesophageal Junction | 33 | 2.06 |
| C 16 | Stomach | 301 | 18.83 |
| C17.0 | Duodenum | 2 | 0.12 |
| C 18 | Colon | 158 | 9.89 |
| C20 | Rectum | 107 | 6.69 |
| C 21 | Anal canal | 3 | 0.19 |
| C 22 | Liver | 12 | 0.75 |
| C 23 | Gall Bladder | 28 | 1.75 |
| C 25 | Pancreas | 17 | 1.06 |
| C30-C39,C11,C12 | **Respiratory System** | **234** | **14.64** |
| C 11 | Nasopharynx | 10 | 0.62 |
| C 30 | Nasal cavity | 6 | 0.37 |
| C 31 | Para nasal sinus | 1 | 0.06 |
| C 32 | Larynx | 73 | 4.57 |
| C 34 | Lung | 144 | 9.01 |
| C40-C41 | **Bones, Joints and articular Cartilages** | **100** | **6.26** |
| C 40.0 | Maxilla | 11 | 0.69 |
| C 40-41 | Bones (NOS), Femur,Tibia | 89 | 5.57 |
| C50 | **Breast** | **110** | **6.88** |
| C51-C58 | **Female Genital Organs** | **137** | **8.57** |
| C 52 | Vagina | 2 | 0.12 |
| C 53 | Cervix Uteri | 41 | 2.56 |
| C 55 | Corpus Uteri | 27 | 1.69 |
| C 56 | Ovary | 67 | 4.19 |
| C60-C63 | **Male Genital Organs** | **26** | **1.63** |
| C 61 | Prostate | 11 | 0.69 |
| C62 | Testis | 15 | 0.94 |
| C64-C68 | **Urinary tract** | **64** | **4.00** |
| C 64 | Kidney | 20 | 1.25 |
| C 67 | Urinary Bladder | 44 | 2.75 |
| C81- C96 | **Lymphoid and Haemopiotic System** | **56** | 3.50 |
| C95,C91,C92 | Leukemias (NOS), ALL,CLL,CML,Multiple Myeloma | 27 | 1.69 |
| C81,C82-C85 | Lymphomas(NOS), Hodgkins and Non Hodgkins Lymphomas | 29 | 1.81 |
| C 44- C44.9 | **Skin** | **41** | 2.56 |
| C 47.2 | **Nerves** | **3** | 0.19 |
| C48 | **Retroperitoneum** | **5** | 0.31 |
| C 49 | **Soft tissue** | **6** | 0.37 |
| **C69-C72** | **CNS** | **6** | 0.37 |
| C 69.2 | Retina | 3 | 0.19 |
| C 71 | Brain | 3 | 0.19 |
| C73-C75 | **Thyroid and other Endocrine Glands** | **76** | 4.75 |
| C 73 | Thyroid | 73 | 4.57 |
| C 74 | Adrenal gland | 1 | 0.06 |
| C 75.1 | Pituitary gland | 1 | 0.06 |
| C 75.4 | Carotid Body | 1 | 0.06 |
| C 80.9 | **Unknown primary site** | 5 | 0.31 |
| **All Sites** |  | **1598** | 100 |
